# Supplementary material for: Nrf2 regulates ICAM-1–mediated neutrophil extracellular trap formation after traumatic brain injury
Source: bioRxiv. 2026 May 6:2026.05.01.722360. Preprint. [Version 1] doi: 10.64898/2026.05.01.722360 (PMC13174394; doi:10.64898/2026.05.01.722360)
Supplement: 1 [file NIHPP2026.05.01.722360V1-supplement-1.pdf]

bioRxiv preprint doi: https://doi.org/10.64898/2026.05.01.722360; this version posted May 6, 2026. The copyright holder for this preprint (which was not certified by peer review) is the author/funder, who has granted bioRxiv a license to display the preprint in perpetuity. It is made available under aCC-BY 4.0 International license.

Nrf2 preserves neurovascular integrity and promotes functional recovery by limiting leukocyte transmigration and neutrophil extracellular trap formation in traumatic brain injury.

P. M. Abdul Muneer[1]*, Saurav Bhowmick[2], Yemin A. Poovanthodi[2], Saleena Alikunju[3]

[1]Laboratory of CNS Injury and Molecular Therapy, Department of Biomedical Engineering, Florida International University, 10555 West Flagler Street, Miami, FL-33174.

[2]JFK Neuroscience Institute, Hackensack Meridian Health JFK University Medical Center, 65 James St, Edison, NJ-08820, United States.

[3]Miami Project to Cure Paralysis, Lois Pope LIFE Center, Miller School of Medicine, University of Miami, 1095 NW 14th Terrace, Miami, FL 33136, United States.

Running title: Nrf2 in leukocyte transmigration and NET formation after TBI.

bioRxiv preprint doi: https://doi.org/10.64898/2026.05.01.722360; this version posted May 6, 2026. The copyright holder for this preprint (which was not certified by peer review) is the author/funder, who has granted bioRxiv a license to display the preprint in perpetuity. It is made available under aCC-BY 4.0 International license.

Supplemental Materials:

Supplemental Figures:

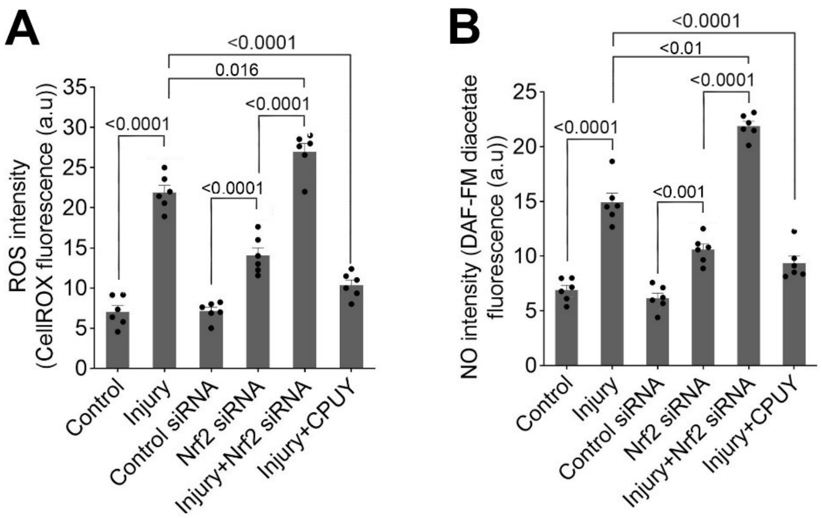

Supplementary Figure 1: Measurement of ROS and NO in stretch-injured hBMVECs. (**G**) Representative images and quantification of intracellular reactive oxygen species (ROS) levels measured using CellROX Green fluorescence in control and stretch-injured hBMVECs with control siRNA, Nrf2 siRNA or CPUY192018 treatment (n = 6/group).
(**H**) Quantification of nitric oxide (NO) production assessed by DAF-FM diacetate fluorescence in control and stretch-injured hBMVECs with control siRNA, Nrf2 siRNA or CPUY192018 treatment (n = 6/group).
All values are expressed as mean ± SEM. Statistical analysis was performed using one-way ANOVA in B-C and G-H or two-way ANOVA in E-F, followed by Dunnett's post hoc test. $p < 0.05$ statistically significant.

bioRxiv preprint doi: https://doi.org/10.64898/2026.05.01.722360; this version posted May 6, 2026. The copyright holder for this preprint (which was not certified by peer review) is the author/funder, who has granted bioRxiv a license to display the preprint in perpetuity. It is made available under aCC-BY 4.0 International license.

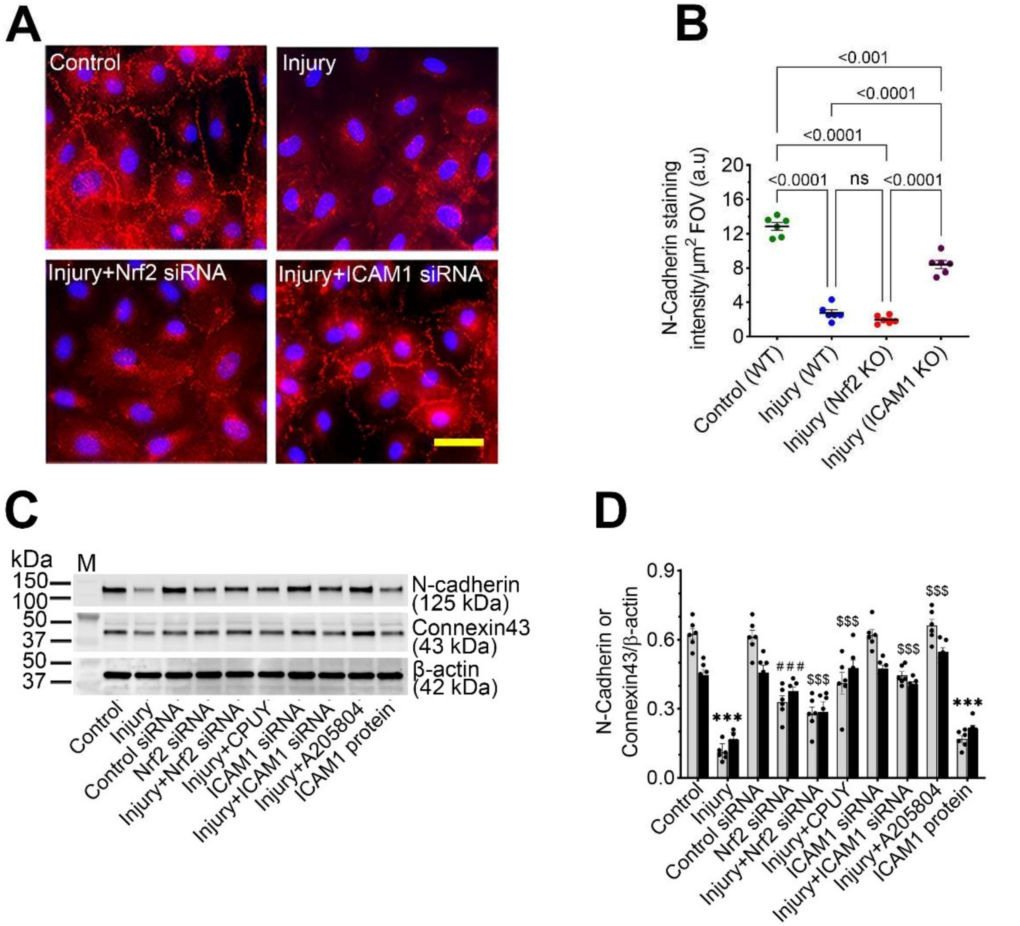

Supplementary Figure 2. Nrf2 and ICAM-1 regulate junctional protein expression following *in vitro* stretch injury.

(**A-B**) Representative immunofluorescence staining (A) and quantification (B) of N-cadherin (red) with nuclear counterstain DAPI (blue) in hBMVEC following 3.0 psi stretch injury with or without Nrf2 or ICAM-1 siRNA treatment (n = 6/group). Scale bar = 40 µm.

(**C-D**) Western blot analysis of N-cadherin, Connexin-43, and β-actin in lysates from stretch-injured hBMVEC cultures 24 h after treatment with control siRNA, Nrf2 siRNA, the Nrf2 activator CPUY, ICAM-1 siRNA, the ICAM-1 inhibitor A2015804, or recombinant ICAM-1 protein. Bar graphs represent densitometric quantification of N-cadherin (D) normalized to β-actin (n = 6/group).

All values are expressed as mean ± SEM. Statistical analysis was performed using one-way ANOVA in B and D followed by Dunnett's post hoc test.

p < 0.05 statistically significant.

$^{***}P < 0.001$ versus control; $^{\#\#\#}P < 0.001$ versus control siRNA; $^{\$\$\$}P < 0.001$ versus injury in D. 'ns' is not significant.

bioRxiv preprint doi: https://doi.org/10.64898/2026.05.01.722360; this version posted May 6, 2026. The copyright holder for this preprint (which was not certified by peer review) is the author/funder, who has granted bioRxiv a license to display the preprint in perpetuity. It is made available under aCC-BY 4.0 International license.

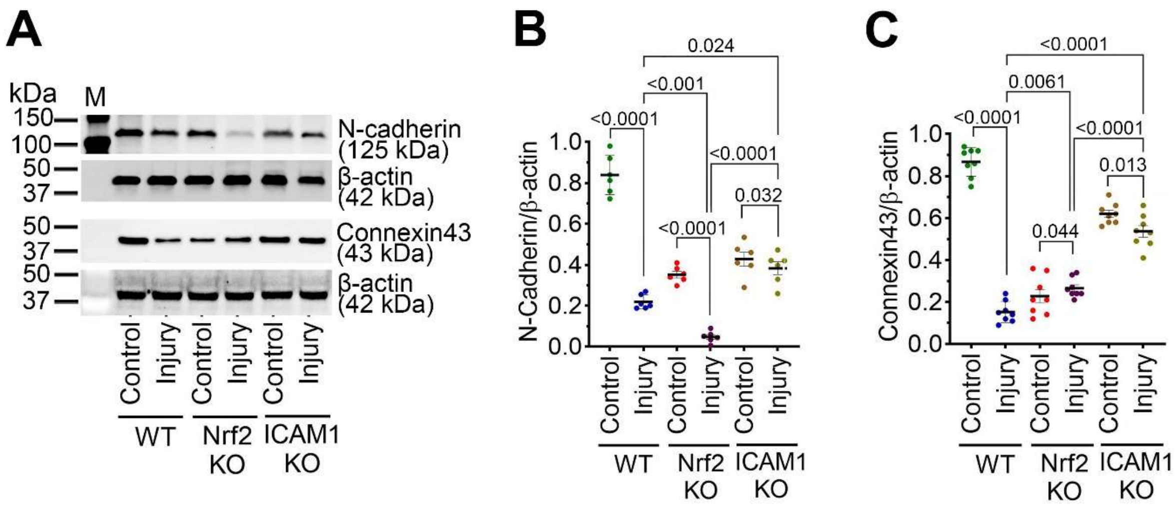

Supplementary Figure 3. Nrf2 and ICAM-1 regulate junctional protein expression following *in vivo* injury.

(**A-C**) Western blot analysis of N-cadherin and Connexin-43 with β-actin as a loading control in cortical brain tissue lysates from WT, *Nrf2* $^{-/-}$, and *ICAM-1* $^{-/-}$ mice following 15 psi FPI. Bar graphs show densitometric quantification of N-cadherin (F) and connexin-43 (G) normalized to β-actin (n = 6/group).

All values are expressed as mean ± SEM. Statistical analysis was performed using two-way ANOVA in B and C followed by Dunnett's post hoc test.

$p < 0.05$ statistically significant.

$^{***}P < 0.001$ versus control; $^{\#\#\#}P < 0.001$ versus control siRNA; $^{\$\$\$}P < 0.001$ versus injury in D.

Statistical significance between groups is indicated on the graphs B and C.

bioRxiv preprint doi: https://doi.org/10.64898/2026.05.01.722360; this version posted May 6, 2026. The copyright holder for this preprint (which was not certified by peer review) is the author/funder, who has granted bioRxiv a license to display the preprint in perpetuity. It is made available under aCC-BY 4.0 International license.

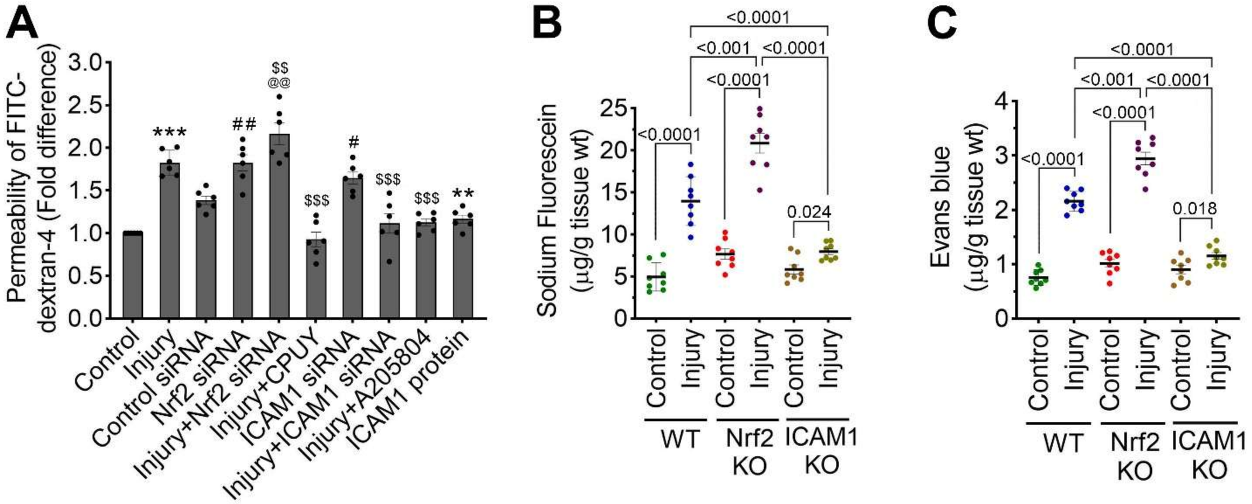

Supplemental Figure 4. Nrf2 regulates BBB integrity through ICAM-1 following traumatic injury.

(**A**) *In vitro* assessment of blood–brain barrier (BBB) integrity using FITC-dextran permeability assay in stretch-injured hBMVEC cultures 24 h after treatment with control siRNA, Nrf2 siRNA, the Nrf2 activator CPUY, ICAM-1 siRNA, the ICAM-1 inhibitor A2015804, or recombinant ICAM-1 protein. Bar graphs represent fold change quantification of FITC-dextran-4 permeability normalized to control group (n = 6/group).

(**B-C**) *In vivo* evaluation of BBB permeability following injury using sodium fluorescein (B) and Evans blue (**C**) tracer assays in wild-type (WT), *Nrf2* $^{-/-}$, and *ICAM-1* $^{-/-}$ mice (n = 8/group).

All values are expressed as mean ± SEM. Statistical analysis was performed using one-way ANOVA in A or two-way ANOVA in B and C followed by Dunnett's post hoc test.

$p < 0.05$ statistically significant.

$^{**}P < 0.01$, $^{***}P < 0.001$ versus control; $^{\#}P < 0.05$, $^{\#\#}P < 0.01$ versus control siRNA; $^{@@}P < 0.01$ versus Nrf2 siRNA; $^{\$\$}P < 0.01$, $^{\$\$\$}P < 0.001$ versus injury in A. Statistical significance between groups is indicated on the graphs B and C.

bioRxiv preprint doi: https://doi.org/10.64898/2026.05.01.722360; this version posted May 6, 2026. The copyright holder for this preprint (which was not certified by peer review) is the author/funder, who has granted bioRxiv a license to display the preprint in perpetuity. It is made available under aCC-BY 4.0 International license.

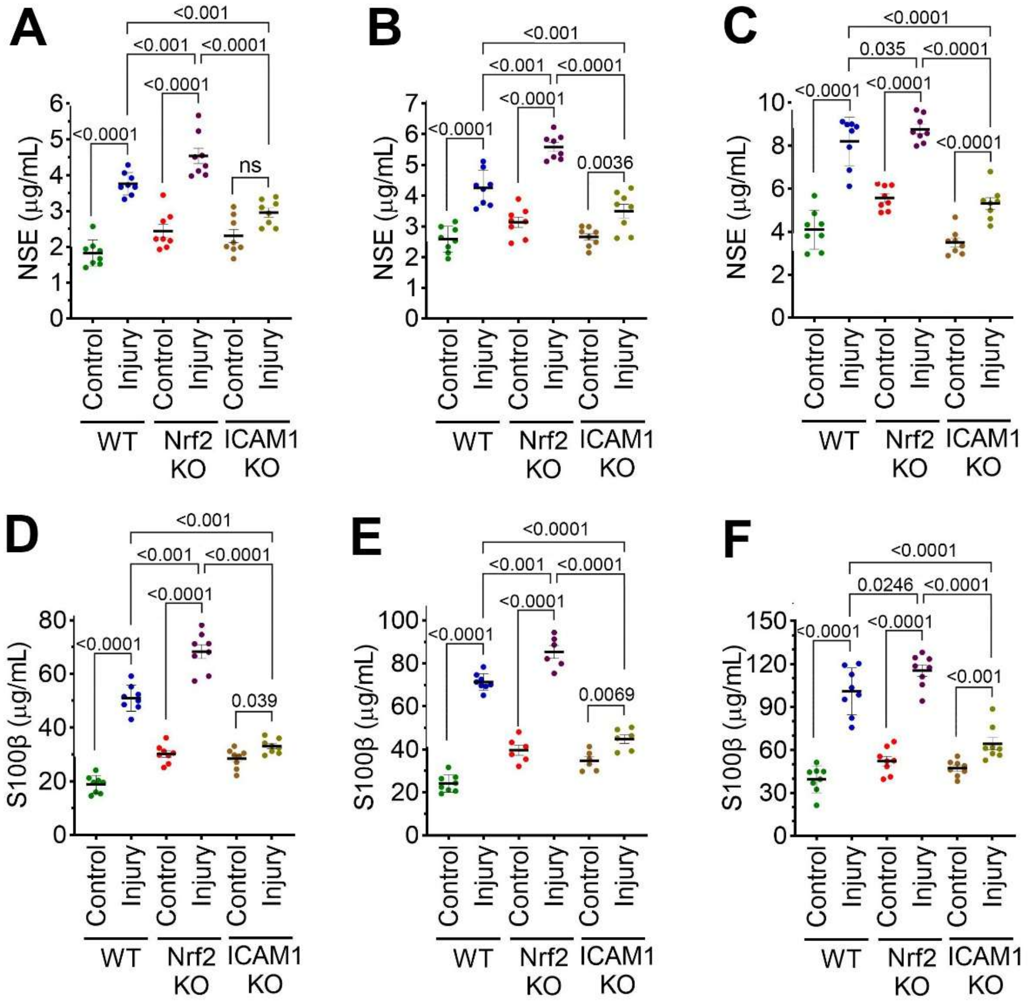

Supplemental Figure 5. Injury-associated changes in S100β and NSE levels across brain tissue, plasma, and CSF.

(**A-C**) Quantification of neuron-specific enolase (NSE) levels in brain tissue lysate (A), blood plasma (B), and cerebrospinal fluid (CSF) (C) from wild-type (WT), *Nrf2*$^{-/-}$, and *ICAM-1*$^{-/-}$ mice under control and injury conditions (n = 8/group).

(**D-F**) Quantification of S100β levels in brain tissue lysate (D), blood plasma (E), and CSF (F) across the same experimental groups (n = 8/group).

All values are expressed as mean ± SEM. Statistical analysis was performed using one-way ANOVA in A or two-way ANOVA in B and C followed by Dunnett's post hoc test. Statistical significance between groups is indicated on the graphs. $p < 0.05$ statistically significant.

bioRxiv preprint doi: https://doi.org/10.64898/2026.05.01.722360; this version posted May 6, 2026. The copyright holder for this preprint (which was not certified by peer review) is the author/funder, who has granted bioRxiv a license to display the preprint in perpetuity. It is made available under aCC-BY 4.0 International license.

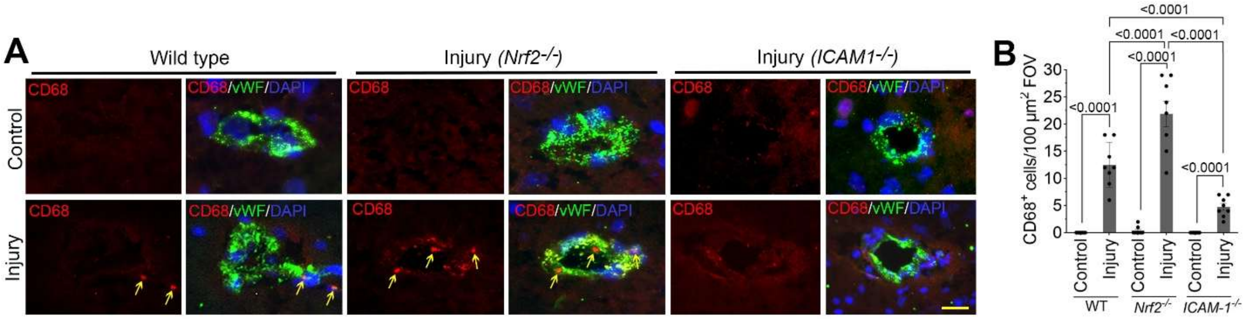

Supplemental Figure 6. Nrf2 and ICAM-1 regulate macrophage infiltration following in vivo injury.

(**A-B**) Immunohistochemical analysis of $CD68^{+}$ macrophage (red), and co-localized with von Willibrand Factor (vWF, a microvessel maker, green) and DAPI (nucleus, blue). Infiltration of $CD68^{+}$ cells into the perivascular space in wild-type (WT), *Nrf2*$^{-/-}$, and *ICAM-1*$^{-/-}$ mice under control and injury conditions are shown in the representative images (A) and corresponding quantification (B) (n = 8/group). Scale bar = 40 μm.

All values are expressed as mean ± SEM. Statistical analysis was performed using two-way ANOVA followed by Dunnett's post hoc test and statistical significance is indicated on the graph. $p < 0.05$ statistically significant.

bioRxiv preprint doi: https://doi.org/10.64898/2026.05.01.722360; this version posted May 6, 2026. The copyright holder for this preprint (which was not certified by peer review) is the author/funder, who has granted bioRxiv a license to display the preprint in perpetuity. It is made available under aCC-BY 4.0 International license.

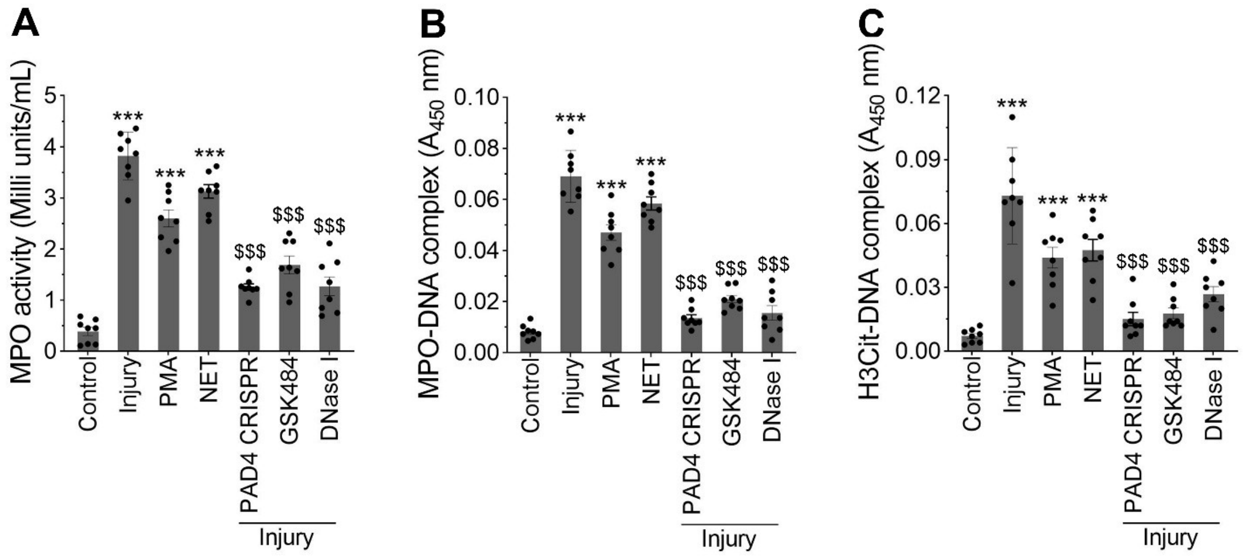

Supplemental Figure 7. Activation of NET-associated effector pathways in hBMVECs following injury.

(**A**) Measurement of myeloperoxidase (MPO) activity in human brain microvascular endothelial cells (hBMVECs) under control conditions, injury, and indicated treatments, including PMA stimulation, purified NET exposure, PAD4 CRISPR-mediated inhibition, GSK484 treatment, and DNase I treatment (n = 8/group).

(**B**) Quantification of MPO-DNA complex formation as an indicator of neutrophil extracellular trap (NET) activity across experimental groups (n = 8/group).

(**C**) Quantification of citrullinated histone H3 (H3Cit)-DNA complexes in the same conditions (n = 8/group).

Data are presented as individual data points with mean ± SEM. Statistical analysis was performed using two-way ANOVA followed by Dunnett's post hoc test and statistical significance is indicated on the graphs.

bioRxiv preprint doi: https://doi.org/10.64898/2026.05.01.722360; this version posted May 6, 2026. The copyright holder for this preprint (which was not certified by peer review) is the author/funder, who has granted bioRxiv a license to display the preprint in perpetuity. It is made available under aCC-BY 4.0 International license.

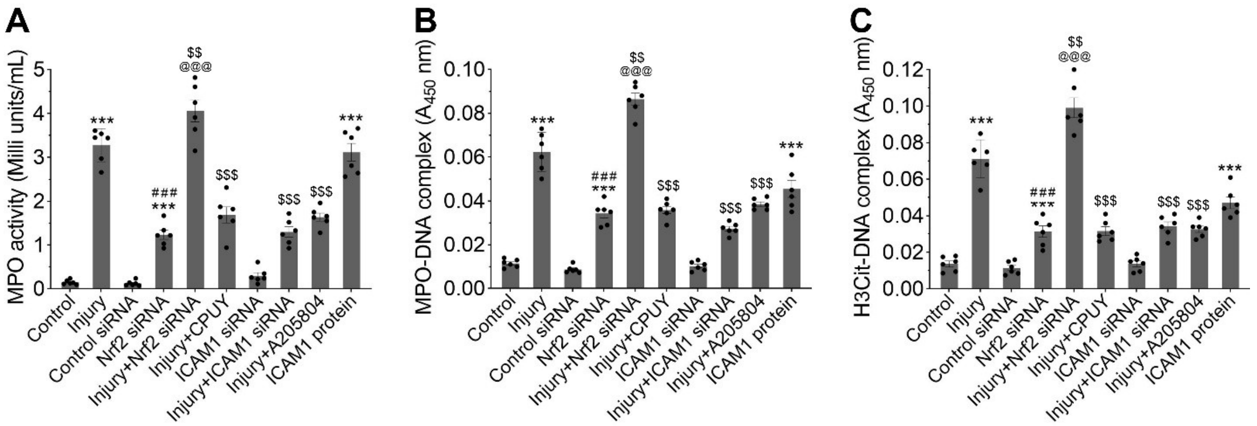

Supplemental Figure 8. Nrf2 and ICAM-1 regulate NET formation following injury in hBMVECs.

(**A**) Measurement of myeloperoxidase (MPO) activity in human brain microvascular endothelial cells (hBMVECs) under control and injury conditions, with Nrf2 knockdown (siRNA), Nrf2 activation (CPUY192018), ICAM-1 knockdown (siRNA), ICAM-1 inhibition (A205804), and recombinant ICAM-1 protein treatment (n = 6/group).

(**B**) Quantification of MPO-DNA complex formation across the indicated experimental conditions (n = 6/group).

(**C**) Quantification of citrullinated histone H3 (H3Cit)-DNA complexes as a marker of neutrophil extracellular trap (NET) formation (n = 6/group).

All values are expressed as mean ± SEM. Statistical analysis was performed using one-way ANOVA followed by Dunnett's post hoc test and statistical significance between groups is indicated on the graphs. $p < 0.05$ statistically significant.

bioRxiv preprint doi: https://doi.org/10.64898/2026.05.01.722360; this version posted May 6, 2026. The copyright holder for this preprint (which was not certified by peer review) is the author/funder, who has granted bioRxiv a license to display the preprint in perpetuity. It is made available under aCC-BY 4.0 International license.

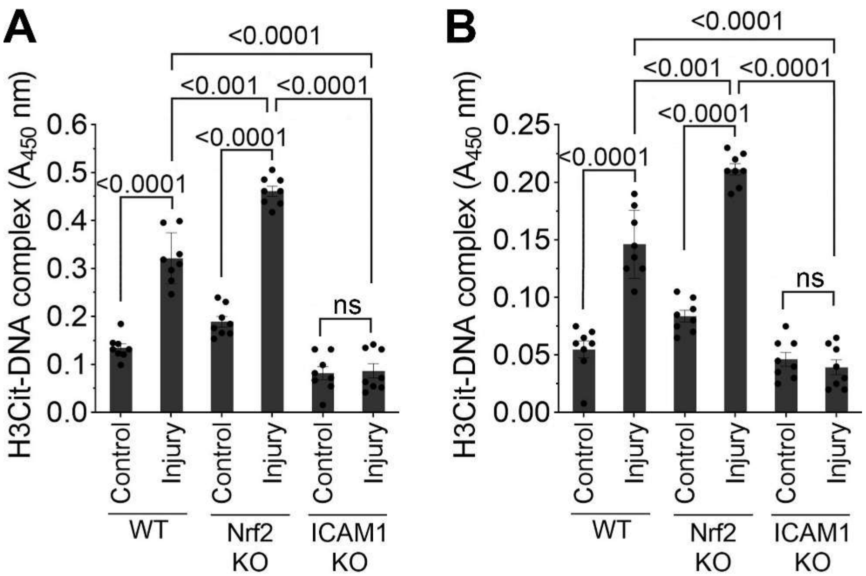

Supplemental Figure 9. Nrf2 and ICAM-1 regulate circulating and tissue NET formation following *in vivo* injury.

(**A-B**) Quantification of citrullinated histone H3 (H3Cit)-DNA complexes by ELISA in brain tissue lysates (A) and plasma samples (B) from wild-type (WT), *Nrf2* $^{-/-}$, and *ICAM-1* $^{-/-}$ mice under control and injury conditions (n = 8/group).

All values are expressed as mean ± SEM. Statistical analysis was performed using two-way ANOVA followed by Dunnett's post hoc test and statistical significance between groups is indicated on the graphs. $p < 0.05$ statistically significant.

bioRxiv preprint doi: https://doi.org/10.64898/2026.05.01.722360; this version posted May 6, 2026. The copyright holder for this preprint (which was not certified by peer review) is the author/funder, who has granted bioRxiv a license to display the preprint in perpetuity. It is made available under aCC-BY 4.0 International license.

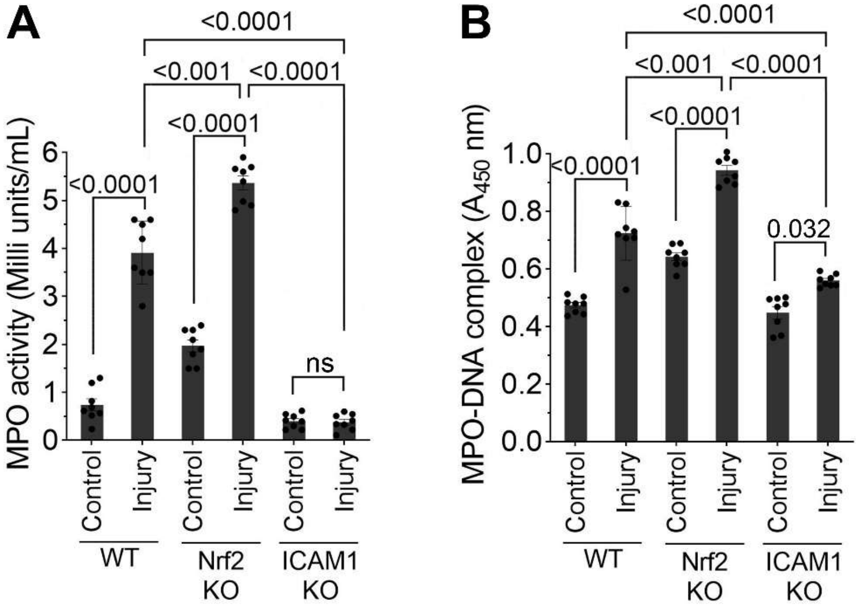

Supplemental Figure 10. Nrf2 and ICAM-1 regulate MPO activity and NET-associated MPO release following *in vivo* injury.

(**A**) Measurement of myeloperoxidase (MPO) activity in brain tissue lysates from wild-type (WT), *Nrf2*$^{-/-}$, and *ICAM-1*$^{-/-}$ mice under control and injury conditions (n = 8/group).

(**B**) Quantification of MPO-DNA complexes in plasma samples as an indicator of NET-associated MPO release across the same experimental groups (n = 8/group).

All values are expressed as mean ± SEM. Statistical analysis was performed using two-way ANOVA followed by Dunnett's post hoc test and statistical significance between groups is indicated on the graphs. $p < 0.05$ statistically significant.

bioRxiv preprint doi: https://doi.org/10.64898/2026.05.01.722360; this version posted May 6, 2026. The copyright holder for this preprint (which was not certified by peer review) is the author/funder, who has granted bioRxiv a license to display the preprint in perpetuity. It is made available under aCC-BY 4.0 International license.

Reagents and Tools Table

Reagent/Resource	Reference or Source	Identifier or Catalog Number
**Experimental Models**		
C57BL/6J WT mice (*M. musculus*)	Jackson Laboratory, Bar Harbor, ME	Strain No.:000664
Nrf2 Knockout mice	Jackson Laboratory, Bar Harbor, ME	B6.129X1-Nfe2l2^{tm1Ywk}/J.
ICAM-1 knockout mice	Jackson Laboratory, Bar Harbor, ME	B6.129S7-*Icam1*^{tm1Bay}/J
GFP transgenic mice	Jackson Laboratory, Bar Harbor, ME	Tg(CAG-EGFP)131Osb/Ley SopJ;
**Recombinant DNA**		
pGL3-GSTm1-Luc promoter reporter plasmids		
pGL3-GPx1-Luc promoter reporter plasmids		
pGL3-HO-1-Luc promoter reporter plasmids		
pGL3-NQO1-Luc promoter reporter plasmids		
pRL-TK Renilla luciferase plasmid	(Promega)	
**Antibodies**		
HRP-conjugated goat secondary antibody		
Anti-β-actin	Thermo Fisher	MA575739
anti-Nrf2	R and D	MAB3925
anti-p-Nrf2	Novus Biologicals	PA5-67520
anti-GPx1	Thermo Fisher	PA5-30593
anti-GSTm1	Thermo Fisher	PA5-22278
anti-HO-1	Gene Tex	GTX101147
anti-NQO1	Abcam	ab80588
anti-NOX1	Sigma- Aldrich	SAB4200097
anti-4HNE	Abcam	ab46545
anti-iNOS	Abcam	ab3523
anti-3NT	Millipore	06-284
anti-ICAM-1	Thermo Fisher	MA5407
anti-Mac1	Novus Biologicals	nb11089474
anti-LFA 1	Abcam	ab186873
anti-MMP-2	Cell Signaling	87809S
anti-MMP-9	Abcam	ab76003
anti-occludin	Abcam	ab31721
anti-claudin-5	Abcam	ab15106
anti-ZO-1	Abcam	ab59720
anti-N-cadherin	Abcam	ab18203
anti-connexin-43	Cell Signaling	3512S
anti-CD68	Abcam	ab955
anti-vWF	Abcam	Ab11713
anti-Ly6G	Cell Signaling	87048S

bioRxiv preprint doi: https://doi.org/10.64898/2026.05.01.722360; this version posted May 6, 2026. The copyright holder for this preprint (which was not certified by peer review) is the author/funder, who has granted bioRxiv a license to display the preprint in perpetuity. It is made available under aCC-BY 4.0 International license.

anti-H3Cit	Cayman Chemicals	17939
anti-H3	Cell Signaling	9715S
anti-PAD4	Thermo Fisher	PA5-22317
**Oligonucleotides and other sequence-based reagents**		
The PAD4 CRISPR All-in-one AAV virus (with saCas9) (Mouse; Serotype9)	Applied Biological Materials, Richmond, BC, Canada.	
Scrambled CRISPR/Cas9 AAV	Applied Biological Materials, Richmond, BC, Canada.	K079
Nrf2 siRNA	Santa Cruz Biotechnology	SC37049
ICAM-1 siRNA	Santa Cruz Biotechnology	SC29354
Scrambled control siRNA	Santa Cruz Biotechnology	SC37007
**Chemicals, Enzymes and other reagents**		
DMEM/F-12 medium	Gibcon, CA, USA	
HEPES		
Sodium bicarbonate		
L-glutamine		
Penicillin+Streptomycin		
Heparin		
Endothelial cell growth supplement	BD Biosciences	
Rat-tail collagen		
CPUY192018	AOBIOUS	AOB9974
A205804	Cayman Chemical	21252
Cranioplastic dental cement	AM Systems, Carlsborg, WA	525000
CellROX Green Reagent Kit	Thermo Fisher Scientific, Rockford, IL	C10444
DAF-FM diacetate Kit	Thermo Fisher Scientific, Rockford, IL	D-23844
Parafolmaldehyde		
Peroxidase substrate reagent (SK-4600; Vector Laboratories)		
CellLytic-M buffer	Thermo Fisher Scientific, Rockford, IL	
DAPI		
Protein A/G magnetic beads		
iTaq Universal SYBR Green Supermix	Bio-Rad Laboratories, USA	
Cell lytic buffer,	Sigma)	
DNA/protein A-agarose magnetic beads		
RNase A		
proteinase K		
Calcein-AM	Invitrogen	
FITC-labelled Dextran-4	Invitrogen,	

bioRxiv preprint doi: https://doi.org/10.64898/2026.05.01.722360; this version posted May 6, 2026. The copyright holder for this preprint (which was not certified by peer review) is the author/funder, who has granted bioRxiv a license to display the preprint in perpetuity. It is made available under aCC-BY 4.0 International license.

Macrophage-colony stimulating factor (MCSF)		
Sodium fluorescein (Na-Fl)		
Evan's Blue (EB)		
Trichloroacetic acid (TCA)		
NaOH		
**Software**		
ImageJ		
GraphPad Prism V9		
**Other**		
Human brain microvascular endothelial cells (hBMVECs)	Cell Biologicals, Chicago, IL.	H-6023.
Human neutrophils	Stemcell Technologies, Cambridge, MA	200-0384
Bicinchoninic acid (BCA) assay	Thermo Fisher Scientific, Rockford, IL	
Pierce™ Classic Magnetic IP/Co-IP Kit	Thermo Fisher Scientific, Rockford, IL	88804.
iScript cDNA Synthesis Kit	Bio-Rad Laboratories, USA	
S100β ELISA Kit	Abnova, Littleton CO, USA	
Neuron specific enolase (NSE) ELISA Kit	Alpha Diagnostic, San Antonio, Texas, USA;	0050

bioRxiv preprint doi: https://doi.org/10.64898/2026.05.01.722360; this version posted May 6, 2026. The copyright holder for this preprint (which was not certified by peer review) is the author/funder, who has granted bioRxiv a license to display the preprint in perpetuity. It is made available under aCC-BY 4.0 International license.

Supplementary Table 2: Primers designed for RT-qPCR expression assays and ChIP-qPCR.

**q-PCR primers:**		
Gene target	FWD primer sequence (5'→ 3')	REV primer sequence (5'→ 3')
Nrf2	GCCTTACTCTCCCAGTGAATAC	CTCCCAAATGGTGCCTAAGA
GPx1	GATCTCAGCACCATCCAGTT	GGACAGCAGGGTTTCTATGT
GSTm1	CGCTACATCGCAACACCTAT	GGGTAATTCTAGGAAGCGTGAG
HO-1	CTCCCTGTGTTTCCTTTCTCTC	CAGTCGTGGTCAGTCAACAT
NQO1	AGTGCTCGTAGCAGGATTTG	TCTGGTTGTCAGCTGGAATG
GAPDH	GGTCGGTGTGAACGGATTT	GTGGATGCAGGGATGATGTT
**ChIP-qPCR primers:**		
Gene target	FWD primer sequence (5'→ 3')	REV primer sequence (5'→ 3')
GPx1	ACA ATA TAA GGG AGC TGT GCG T	CTA GGG CGG GTC TGG TCT A
GSTm1	GGA CAA AGA AAA GGT GGT ACG	TGG GTT AAC TCA CCC AGA ATG
HO-1	TGA AGT TAA AGC CGT TCC GG	AGC GGC TGG AAT GCT GAG T
NQO1	TCT AAG AGC AGA ACG CAG CA	TTC GTG GGA CCT GCC TAC AT
